# Malarial Hematuria

**Published:** 1895-12

**Authors:** J. J. Kilpatrick

**Affiliations:** Midville, Ga.


### *CORRESPONDENCE.*

### MALARIAL HEMATURIA.

MIDVILLE, GA., October 30, 1895.

*Atlanta Medical and Surgical Journal:*

In this short article I wish to give my experience with malarial hematuria (or the so-called hemorrhagic fever). In doing so I will try to state facts as I have found them. I am young in the practice. I have been practicing not quite five years; my work being in a malarial section. I have treated thirty-seven cases, both male and female, ranging in age from four to ninety years, and have not lost a single case. After a thorough study of this dangerous malady, I find the real cause of it to be malaria, and *in this section* it is confined strictly to the white race, as I have never known a negro to have it. At the onset I find the symptoms to be as follows: patient is first taken with a chill, not very decided in every case, and of short duration; he may be attending to his duties, not necessarily confined to his bed. At the expiration of these chilly sensations there is a slight rise of temperature, ranging from 101° to 103°. About this time he has a desire to micturate, the color of the urine being a dark coffee, or, perhaps, a cherry red, and sometimes very copious. The quantity voided is half pint more or less, and often less. Patient does not seem to experience any pain during the voiding of this urine, other than a dead, aching sensation in the region of the kidneys. I have submitted this fluid to a microscopical test and find it contains no blood corpuscles, though I have not subjected it to a spectroscopic test. I often find, at the onset, intense nausea and vomiting, and at the expiration of six or eight hours, sometimes earlier, I find decided jaundice, and often cold feet. I find the indications are for purgation and diuretics.

For sick stomach I give large quantities of warm water; let the patient drink all he can, distend the stomach with same as much as possible; after a thorough washing out in this way, I then let him sip water very hot to palliate the excited stomach, and place a mustard plaster over same; at the same time I give a warm enema of water one quart, yolk of one egg, spirits turpentine ten drops; place a bottle or jug of warm water to his feet; if vomiting by this time is not controlled, I give half grain of cocain hydrochlorate every half-hour until I give a grain and a half. This should be given in capsules; I do not like the use of morphine or other opiates; neither do I like to give antipyretics, as the heavy purgation and diuretics seem to control the temperature. As a purgative, I give from ten to twenty grains of calomel, with equally as much soda bicarb., every hour to an hour and a half, until I get large, dark green or black stools. I often give, owing to strength of patient, as much as fifty grains of the above every four hours until I give one hundred and fifty grains; at the same time I give, as a diuretic, potassium acetate, two ounces, by measure, to water, three ounces; dissolve well and give tablespoonful in a wineglass or more of water every half to every hour until urine resumes its nornal color. When this is done I feel that my patient is safe. My after-treatment is as follows: Tincture nux vomica and Fowler's solution, equal parts; ten drops three times daily; if patient is very anemic, I add muriate tincture of iron; also after skin clears up, I give quinine bimuriate, grains three to four, three times daily. Of course the above is for adults.

I will just say to the profession, when called to treat this fever, go to your patient as early as possible, as the duration of it is very short and requires prompt and heroic treatment. Stay with your patient, and keep kidneys and bowels acting freely. Possibly you will ask why I do not use quinine. My main reason is that it often keeps the stomach upset, and again it is so slowly assimilated that you have not time to wait upon it. I use no hemostatics, as I find the urine, no matter how red, does not contain any blood corpuscles, provided there are no organic lesions about the kidneys or bladder. I have often discharged patients in from three to five days; let their diet be light, for fear of relapse. I never worry about the temperature or circulation, as they will take care of themselves, if you will rid the system of this engorgement of malaria. You may then ask what gives the urine this color. As I have said, it contains no blood corpuscles; I find it to be hemoglobin and bile pigment.

I have given you in the foregoing, as best I could, all that I know about this fever, and hope it will be the means of helping some brother who has not had as much experience with this malady as I have. J. J. Kilpatrick, M.D.

